# Supplementary material for: Comparative genomics provides new insights into the diversity, physiology, and sexuality of the only industrially exploited tremellomycete: Phaffia rhodozyma
Source: BMC Genomics. 2016 Nov 9;17:901. doi: 10.1186/s12864-016-3244-7 (PMC5103461; doi:10.1186/s12864-016-3244-7)
Supplement: Additional file 6: — List of orphan genes with links to PFAM (related to Additional file 1: Table S1). (ZIP 1428 kb) [file 12864_2016_3244_MOESM6_ESM.zip › PFAM_UNIQUE/G02713_P.html]

#                                                                            --- full sequence --- -------------- this domain -------------   hmm coord   ali coord   env coord
  
# target name        accession   tlen query name           accession   qlen   E-value  score  bias   #  of  c-Evalue  i-Evalue  score  bias  from    to  from    to  from    to  acc description of target
  
#------------------- ---------- ----- -------------------- ---------- ----- --------- ------ ----- --- --- --------- --------- ------ ----- ----- ----- ----- ----- ----- ----- ---- ---------------------
  
G02713\_P             -            974 DUF3299              PF11736.3    146      0.06   11.9   0.0   1   1   2.8e-05      0.17   10.5   0.0    91   140   606   657   604   660 0.87 - Protein of unknown function (DUF3299)  
G02713\_P             -            974 DUF4654              PF15547.1    138     0.016   14.2   0.0   1   5       1.5   2.9e+03   -2.8   0.4    87   104    39    56    13    78 0.56 - Domain of unknown function (DUF4654)  
G02713\_P             -            974 DUF4654              PF15547.1    138     0.016   14.2   0.0   2   5       1.3   2.5e+03   -2.6   2.3    60   113   130   183   121   198 0.62 - Domain of unknown function (DUF4654)  
G02713\_P             -            974 DUF4654              PF15547.1    138     0.016   14.2   0.0   3   5      0.68   1.4e+03   -1.8   0.6    48    97   371   418   334   428 0.64 - Domain of unknown function (DUF4654)  
G02713\_P             -            974 DUF4654              PF15547.1    138     0.016   14.2   0.0   4   5     8e-06     0.016   14.2   0.0    28   133   607   708   602   711 0.83 - Domain of unknown function (DUF4654)  
G02713\_P             -            974 DUF4654              PF15547.1    138     0.016   14.2   0.0   5   5       1.5     3e+03   -2.9   0.4    65    98   786   819   776   859 0.63 - Domain of unknown function (DUF4654)
